# Restraining of glycoprotein VI- and integrin α2β1-dependent thrombus formation by platelet PECAM1

**DOI:** 10.1007/s00018-023-05058-2

**Published:** 2024-01-18

**Authors:** Natalie J. Jooss, Marije G. Diender, Delia I. Fernández, Jingnan Huang, Floor C. J. Heubel-Moenen, Arian van der Veer, Marijke J. E. Kuijpers, Natalie S. Poulter, Yvonne M. C. Henskens, Maroeska te Loo, Johan W. M. Heemskerk

**Affiliations:** 1 Department of Biochemistry, Maastricht University, Maastricht, The Netherlands; 2 Institute of Cardiovascular Sciences, College of Medical and Dental Sciences, University of Birmingham, Birmingham, UK; 3 Department of Pediatric Hematology, Amalia Children’s Hospital, Radboud UMC, Nijmegen, The Netherlands; 4 Platelet Proteomics Group, Centre for Research in Molecular Medicine and Chronic Diseases (CIMUS), Universidad de Santiago de Compostela, Santiago de Compostela, Spain; 5 Department of Internal Medicine, Maastricht University Medical Centre+, Maastricht, The Netherlands; 6 Department of Pediatric Hematology, Maastricht University Medical Center, Maastricht, The Netherlands; 7 Centre of Membrane Proteins and Receptors, Universities of Birmingham and Nottingham, Nottingham, Midlands UK; 8 Central Diagnostic Laboratory, Maastricht University Medical Centre, Maastricht, The Netherlands; 9 Present Address: Synapse Research Institute Maastricht, Kon. Emmaplein 7, 6217 KD Maastricht, The Netherlands; 10 Present Address: Molecular Haematology Unit, University of Oxford, Headington, OX3 9DS UK

**Keywords:** Collagens, PTPN11, SHP2, Noonan syndrome, Microspots, Microfluidics

## Abstract

The platelet receptors, glycoprotein VI (GPVI) and integrin α2β1 jointly control collagen-dependent thrombus formation via protein tyrosine kinases. It is unresolved to which extent the ITIM (immunoreceptor tyrosine-based inhibitory motif) receptor PECAM1 and its downstream acting protein tyrosine phosphatase PTPN11 interfere in this process. Here, we hypothesized that integrin α2β1 has a co-regulatory role in the PECAM1- and PTPN11-dependent restraint of thrombus formation. We investigated platelet activation under flow on collagens with a different GPVI dependency and using integrin α2β1 blockage. Blood was obtained from healthy subjects and from patients with Noonan syndrome with a gain-of-function mutation of *PTPN11* and variable bleeding phenotype. On collagens with decreasing GPVI activity (types I, III, IV), the surface-dependent inhibition of PECAM1 did not alter thrombus parameters using control blood. Blockage of α2β1 generally reduced thrombus parameters, most effectively on collagen IV. Strikingly, simultaneous inhibition of PECAM1 and α2β1 led to a restoration of thrombus formation, indicating that the suppressing signaling effect of PECAM1 is masked by the platelet-adhesive receptor α2β1. Blood from 4 out of 6 Noonan patients showed subnormal thrombus formation on collagen IV. In these patients, effects of α2β1 blockage were counterbalanced by PECAM1 inhibition to a normal phenotype. In summary, we conclude that the suppression of GPVI-dependent thrombus formation by either PECAM1 or a gain-of-function of PTPN11 can be overruled by α2β1 engagement.

**Supplementary Information:**

The online version contains supplementary material available at 10.1007/s00018-023-05058-2.

## Introduction

Glycoprotein VI (GPVI) is a key signaling receptor for collagen in human platelets, acting under flow together with the adhesive collagen receptor integrin α2β1 [1–4]. This set of receptors comes into action when subendothelial collagens are exposed to the blood stream, for instance in vessel wall injury. Platelet adhesion is initiated via collagen-bound von Willebrand factor (VWF), which in a shear-dependent way interacts with the GPIb-V–IX complex [5, 6]. Subsequently both GPVI and integrin α2β1 accomplish activation of the platelets resulting in thrombus formation [7–9]. Platelet GPVI associates with the FcR γ-chain, which carries an immunoreceptor tyrosine-based activation motif (ITAM). The phosphorylation of two tyrosines in the ITAM motif via Src family kinases (SFK) accomplishes a cascade of protein phosphorylation reactions, in particular of the tyrosine kinase Syk, the adapter protein LAT, and the effector proteins phosphatidylinositol 3 kinases (PI3K) and phospholipase C (PLC)γ2 [10–12]. Upon GPVI stimulation, the activated PLCγ2 generates inositol trisphosphate, mediating intracellular Ca^2+^ rises [9]. Downstream functional events are platelet aggregation via integrin αIIbβ3-fibrinogen interaction, granule secretion, P-selectin expression, and surface exposure of the procoagulant phospholipid phosphatidylserine [9, 13]. In this setting, some receptor-type protein tyrosine phosphatases such as CD148 (gene *PTPRJ*) play a positive role by accomplishing initial SFK activation [14].

Other platelet receptors baring the immunoreceptor tyrosine-based inhibitory motif (ITIM) are known to antagonize the signaling of ITAM-linked platelet receptors. This in particular concerns the homotypic activating receptor PECAM1 (platelet-endothelial cell adhesion molecule-1) [15, 16] and the heparin-binding receptor G6b-B (*MPIG6B*).[17–19]. These two ITIM-linked receptors are highly expressed with 9,400 and 13,700 copies per platelet, respectively [20]. It has been established that the inhibitory signaling via ITIM-linked receptors is mediated through protein tyrosine phosphatase non-receptor (PTPN) isoforms, in particular PTPN11 (Src homology region 2 domain-containing phosphatase-2, SHP2) and PTPN22 [21, 22]. On the other hand, different PTPN isoforms appear to enhance rather than suppress ITAM-dependent signaling. This for instance for holds for PTPN1 (protein tyrosine phosphatase 1B, PTP1B) and PTPN6 (SHP1) [21, 23]. The conditions at which platelet ITIM-linked receptors and the associated tyrosine phosphatases can restrain the signaling responses of GPVI are still unclear.

For both human and mouse platelets, there is evidence that gain of function mutations in the *PTPN11* gene restrain specific platelet signaling responses upon GPVI stimulation [24]. Patients with such autosomal dominant mutations are categorized as Noonan syndrome-1, and present with multiple symptoms like a short stature, facial dysmorphism and developmental delay (OMIM: 163,950). In syndromic patients carrying the *PTPN11* mutation, often but not always a moderate bleeding phenotype is observed [25]. The explanation is that the mutated PTPN11 (SHP2) protein in platelets and other cells favors the active conformation, resulting in constitutive tyrosine phosphatase activity and downregulated platelet functions [26, 27]. On the other hand, loss of function mutations in the *PTPN11* gene associate with to the Noonan syndrome with multiple lentigines (NSML) or Leopard syndrome-1 (OMIM: 151,100) without evidence for bleeding.

In the present paper, we hypothesized that, in collagen-dependent thrombus formation, integrin α2β1 has a co-regulatory role with GPVI in the restraint of platelet activation via PECAM1 and PTPN11. We, therefore, set out to investigate under which conditions the blockage of PECAM1 or the modulation of PTPN11 activity led to alterations in thrombus formation on collagen surfaces, inducing platelet adhesion via α2β1 with a differential involvement of GPVI. Rationale for this approach is the expectation that a suppressive effect of PECAM1 signaling is most prominent at low GPVI-induced platelet activation. In sets of experiments, we aimed to untangle this process by inhibiting PECAM1 on collagen-adhered platelets, and we used blood from Noonan patients with a confirmed gain-of-function PTPN11 mutation.

## Methods

### Materials

Human collagen IV (C7521) from Sigma-Aldrich (Zwijndrecht, The Netherlands); human collagen III (1230-01S) from Southern Biotechnology (Birmingham, AL, USA). Blocking 6F1 mAb against integrin α2β1 was a kind gift from Dr. B. S. Coller (Rockefeller University, NY, USA). The inhibitory mouse anti-human PECAM1 mAb (303,101, clone WM59, mouse IgG1) was purchased from BioLegend (London, UK). Control anti-PECAM-1.2 mAb (MABF-2034, clone MBC 78.2, mouse IgG) came from Sigma-Aldrich (Merck, Amsterdam, The Netherlands) Polyclonal anti-G6b-B Ab (PA5-23,300, rabbit IgG), inhibitory anti-FcγRIIA mAb (clone IV.3, mouse IgG2), and mouse IgG1 isotype control came from ThermoFisher Scientific (Eindhoven, The Netherlands). Anti-CD148 mAb (MABC-87, clone Ab1) and D-Phe-Pro-Arg chloromethyl ketone (PPACK) were from Sigma-Aldrich. Fluorescent stains came from the following sources: Alexa Fluor (AF)647-labeled anti-human CD62P mAb (304,918, BioLegend); FITC-labeled anti-fibrinogen antibody (F0111, Dako, Amstelveen, The Netherlands); and AF568-labeled annexin A5 (A13202, ThermoFisher, Eindhoven, The Netherlands). Other materials were from sources described before [28].

### Subjects

Blood donors gave written informed consent according to the declaration of Helsinki to participate in the study. Blood samples were obtained by venipuncture from healthy volunteers, who had not received anti-platelet medication for at least 2 weeks. Approval was obtained from the medical ethics committee from Maastricht University Medical Centre^+^ (MUMC^+^, code: 2017–0285). Permission of blood drawing from Noonan patients was obtained from the Medical Ethics Committees of Radboud University (Evaluation of Bleeding Disorders in Noonan Patients, non-WMO) and MUMC^+^ (ProBe-AHP: Predictors of Bleeding Evaluation in Adult Hematologic Patients with Bleeding Tendencies, METC 14–4-036, Dutch Trial Register, NL9643). For the patient study, blood samples were taken from 13 control subjects, including 6-day controls, and from 6 patients with established Noonan syndrome.

### Collection and preparation of blood samples

Blood was taken from healthy donors or patients into 3.2% trisodium citrate tubes (Vacuette tubes, Greiner Bio-One, Alphen a/d Rijn, The Netherlands). Subjects had normal platelet counts within the reference range, such as established with a Sysmex XN-9000 analyzer (Sysmex, Cho-ku, Kobe, Japan). Platelet count from one patient (NS3) was slightly below the normal range.

### Microfluidics and thrombus formation

Degreased coverslips were coated with 0.5 µL microspots of 100 µg/mL collagen I, collagen III or collagen IV, as described before [13]. Per coverslip, coating was with two spots of the same collagen (humid chamber, overnight 4 °C). After a wash, the downstream microspot was post-coated for 1 h with antibody solution (1 µg/mL). The coated microspots were blocked with Hepes buffer pH 7.45 containing 1% BSA, before mounting into a flow chamber [13].

Prior to perfusion, whole blood samples pre-incubated with vehicle or inhibitor.

(10 min), were supplemented with PPACK (40 µM, f.c.) and recalcified (3.75 mM MgCl_2_ and 7.5 mM CaCl_2_). Samples were then perfused through a parallel plate flow chamber at wall shear rate of 1000 s^−1^ for 3.5 min. Two brightfield images were acquired per microspot, while perfusing the flow chamber with labeling buffer (Hepes buffer pH 7.45 containing 2 mM CaCl_2_, 1 U/mL heparin, and as stains AF647 anti-CD62P mAb, FITC anti-fibrinogen mAb and AF568-annexin A5) [13]. After washing off non-bound label with Hepes buffer, per microspot three additional multicolor fluorescence images were taken [29]. All raw data are given in supplemental datafile.

### Microscopy and image analysis

Images were acquired with an EVOS-FL microscope (Life Technologies, Bleiswijk, The Netherlands), equipped with three fluorescent LEDs combined with dichroic cubes (Cy5, RFP, GFP), an Olympus UPLSAPO 60 × oil-immersion objective, and a sensitive 1360 × 1024-pixel CCD camera. Images were quantified for surface-area-coverage and scored for characteristic thrombus parameters, utilizing semi-automated ImageJ scripts [29]. Parameters 1–5 were generated from brightfield images, and parameters 6–8 from single color fluorescence images (Suppl. Table 1).

### Data handling and statistics

Data were tested for significance using GraphPad Prism V.8 software. Heatmaps were generated with the program R. For heatmap preparation, raw values per blood donor and condition were first averaged and then per parameter univariately scaled (0–10) across surfaces [29]. To visualize treatment effects, subtraction heatmaps of scaled parameters were created to the control values. One-way ANOVA tests were used to assess for statistical significance, set at *p* < 0.05.

## Results

### Blocking of PECAM1 activity restores suppressed collagen-induced thrombus formation by integrin α2β1 blockade

Considering the strong, additive roles of GPVI and integrin α2β1 in thrombus formation on Horm-type collagen [8, 30], we first set out to examine the involvement of both receptors for a range of vascular collagens, i.e. types I, III and IV. The two latter collagens are relatively low in GPVI-induced platelet activation, while collagen III avidly captures VWF (Suppl. Table 1) [31]. For each of the collagens, we studied the platelet activation-modulating role of the ITIM-containing receptor PECAM1, using an anti-PECAM1 mAb (WM59, mouse IgG1), known to abrogate PECAM1-dependent signaling in platelets [32]. The WM59 antibody recognizes the first or second Ig domain in PECAM1, and thereby prevents homophilic ligation of PECAM1 receptors and subsequent tyrosine phosphatase signaling events [16].

To examine the effects of PECAM1 inhibition with collagens, we perfused whole blood samples from healthy subjects over two collagen microspots, of which the downstream microspot was post-coated with inhibitory anti-PECAM1 antibody WM59, under high shear flow conditions [13]. To inhibit integrin α2β1 adhesion, parallel blood samples were pre-incubated with the blocking 6F1 mAb [33]. Platelet thrombi formed on collagens I, III or IV were post-labeled with fluorescent markers for α-granule secretion (AF647 anti-CD62P mAb), integrin αIIbβ3 activation (FITC anti-fibrinogen mAb) and phosphatidylserine exposure (AF659 annexin A5).

**Fig. 1:**
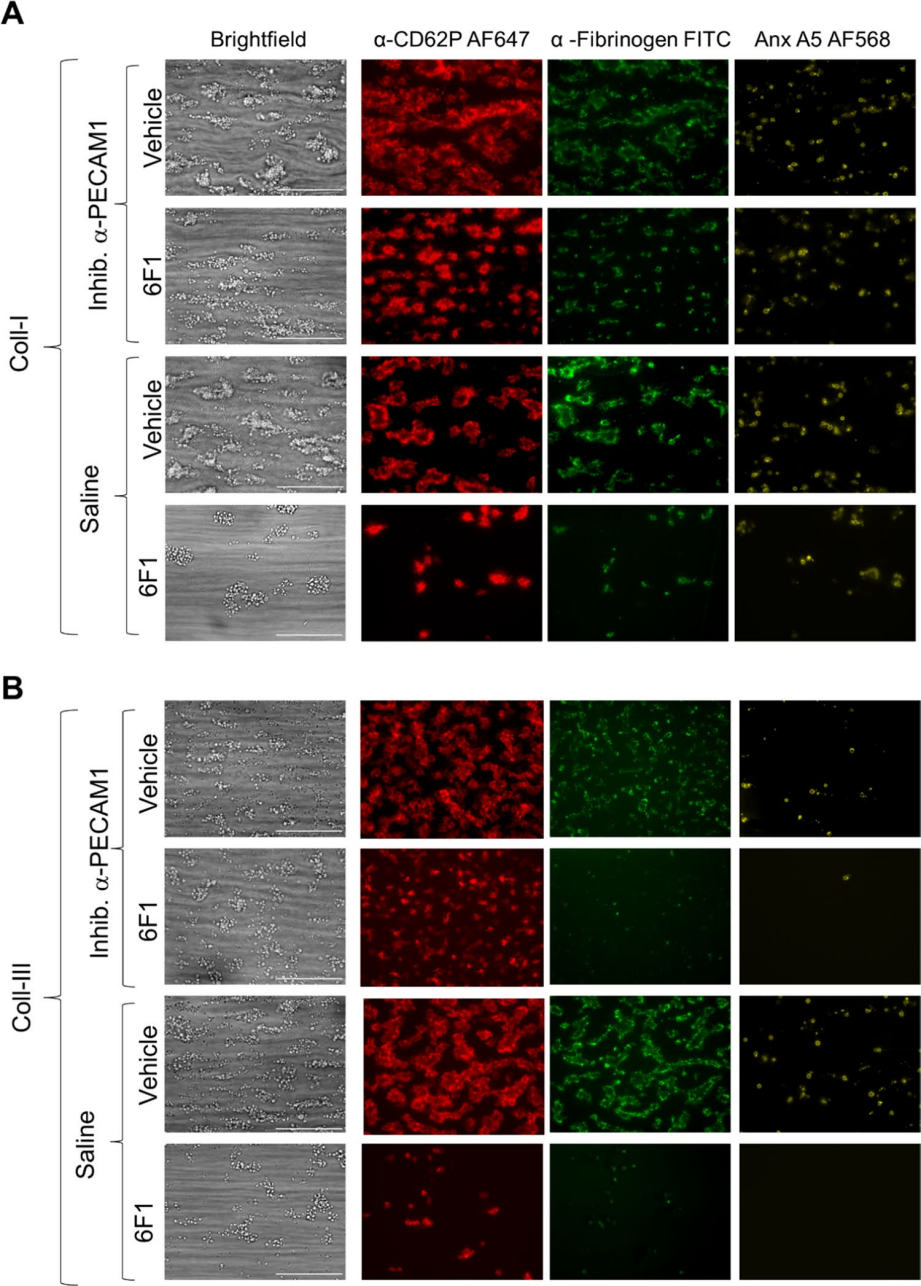
Enhanced collagen-induced thrombus formation by PECAM1 blockage in the absence of integrin α2β1. Blood samples were preincubated with anti-α2β1 6F1 mAb (20 µg/mL) or vehicle, and then perfused during 3.5 min at 1000 s^−1^ over microspots of collagen-I (**A**) or collagen-III (**B**); microspots were post-coated with saline or inhibitory anti-PECAM1 mAb (clone WM59, mouse IgG1). Brightfield and multicolor microscopic images were taken at end stage with an EVOS-FL microscope and 60 × oil objective. Platelet labeling was withAF647 anti-CD62P mAb, FITC anti-fibrinogen mAb and AF658 annexin A5. Shown are representative images for per collagen surface with or without inhibitory anti-PECAM1 mAb (*n* = 6–7). EVOS microscope; scale bars, 50 µm

Under control (vehicle) conditions, large aggregates with activated platelets were formed on collagen I (Fig. 1A). Incubation with 6F1 mAb resulted in substantially smaller thrombi while, interestingly, the presence of inhibitory anti-PECAM1 mAb reverted the effect of 6F1 mAb. When comparing the different surfaces, collagen I, the surfaces collagen III (Fig. 1B) and collagen IV (Suppl. Figure 1C) promoted the formation of smaller sized thrombi with lower platelet activation. On collagen III and especially collagen IV, 6F1 mAb had a strong inhibitory effect, which again was antagonized by co-coated anti-PECAM1 mAb.

**Fig. 2:**
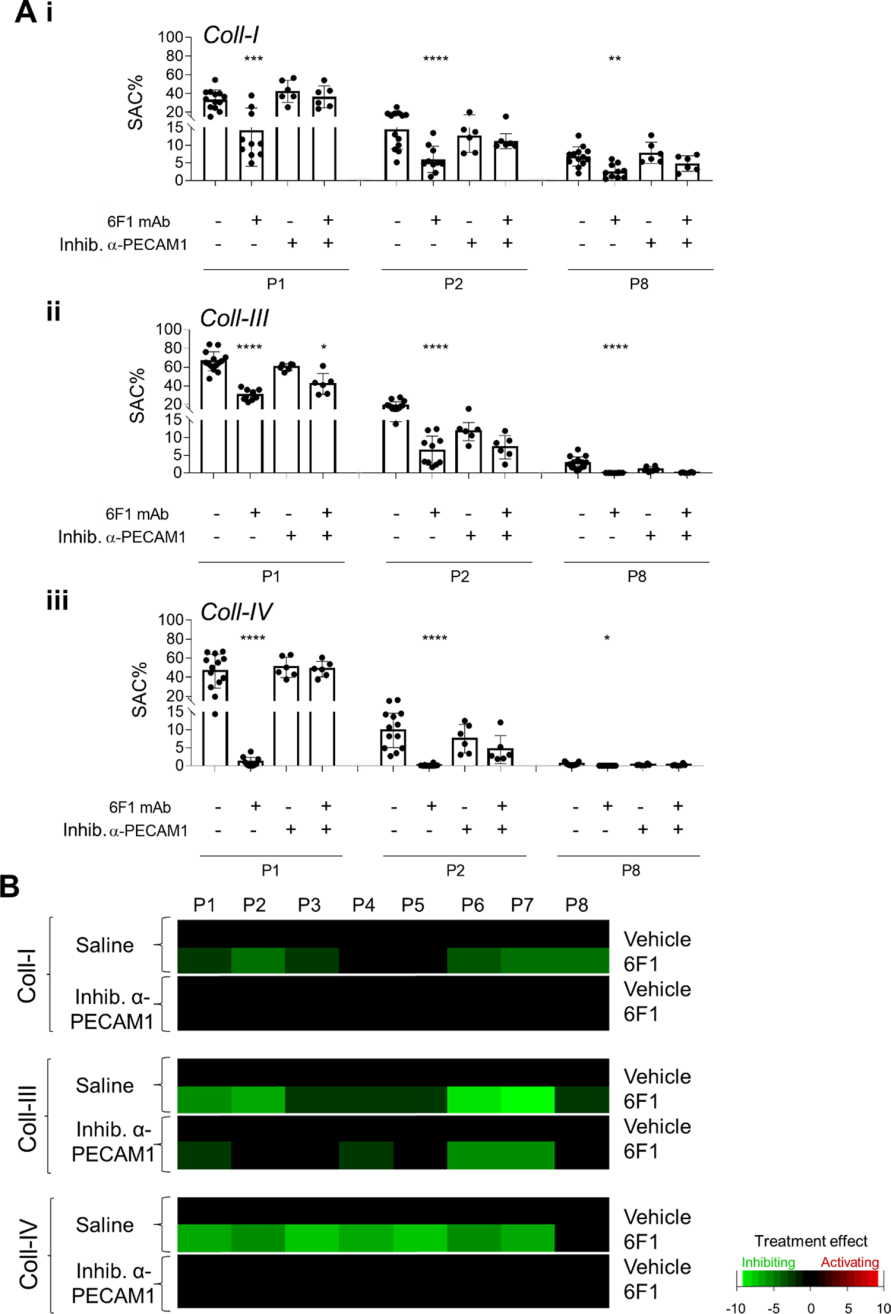
Enhanced collagen-induced thrombus formation by inhibitory anti-PECAM1 antibody upon blockade of integrin α2β1. **A** Blood samples were preincubated with 6F1 mAb (20 µg/mL) or vehicle, and then perfused during 3.5 min at 1000/s over microspots of collagen I (*i*), collagen III (*ii*) or collagen IV (*iii*); microspots were post-coated with saline or inhibitory anti-PECAM1 mAb (WM59 at 1 µg/mL for 1 h). Multicolor microscopic images, as for Fig. 1, were analyzed for platelet adhesion, thrombus phenotype and platelet activation parameters (Suppl. Table 1). Shown are quantified data of platelet deposition (parameter P1), thrombus multilayer size (parameter P2), and phosphatidylserine exposure (parameter P8) in the presence or absence of 6F1 mAb and/or inhibitory anti-PECAM1 mAb. Representative images are shown in Suppl. Fig. 1. **B** Subtraction heatmap presentation of the 8 univariately scaled (0–10) parameters across surfaces, showing values of treatment effect per microspot type relative to vehicle containing blood samples. For the color coding, a relevance filter was applied of *p* < 0.05. Means ± SD (*n* = 6–7); one-way ANOVA *p < 0.05, **p < 0.005, ***p < 0.001, ****p < 0.0001. Raw data of P1-8 are in Suppl. Datafile 1

Quantification of the collected microscopic images provided eight parameters, namely for platelet adhesion (parameter P1), thrombus phenotypes (parameters P2-5), and platelet activation (parameters P6-8), as described before [29]. Analysis (surface-area-coverage) of platelet deposition (P1), thrombus multilayer size (P2) and phosphatidylserine exposure (P8) revealed that α2β1 blockade significantly decreased these parameters for all collagen surfaces (Fig. 2A i–iii). Considering phosphatidylserine exposure as a proxy marker for GPVI activity [30], we noticed a decrease in the order of collagen I > III > IV. Regardless of the collagen type, the presence of anti-PECAM1 mAb alone did not significantly affect platelet adhesion, thrombus size nor platelet procoagulant activity, indicating that the collagen receptor recognition sites were still accessible with the mAb present. In sharp contrast, the anti-PECAM1 mAb co-coating almost fully recovered parameter values (P1, P2, P8) under conditions of α2β1 blockade (Fig. 2A i–iii).

We also generated subtraction heatmaps for all parameters for an integrative comparison of the consequence of PECAM1 inhibition, in cases of absent or present 6F1 mAb. After univariate scaling of the raw data per parameter across all surfaces, values were subtracted versus the vehicle condition, and filtered for relevant effects of interventions (set at *p* < 0.05). The results point to a relevant lowering by 6F1 mAb of 5 (collagen I), 7 (collagen III) and 7 (collagen IV) out of 8 parameters (Fig. 2B). Across all collagen surfaces, the α2β1 blockade most strongly affected thrombus multilayer size (P2), P-selectin expression (P6) and integrin αIIbβ3 activation (P7). Furthermore, with the inhibitory anti-PECAM1 mAb present, reductions by α2β1 blockade were mostly annulled for collagen I and collagen IV, and to a lesser degree for collagen III. Taken together, these data point to a thrombus-stimulating effect of PECAM1 inhibition—i.e. compatible with an ITIM-dependent negative signaling role of PECAM1—only under conditions of α2β1 blockade, regardless of the level of GPVI-dependent platelet activation by the collagen type.

### Selective rescuing effect by inhibitory anti-PECAM1 antibody on thrombus formation

**Fig. 3:**
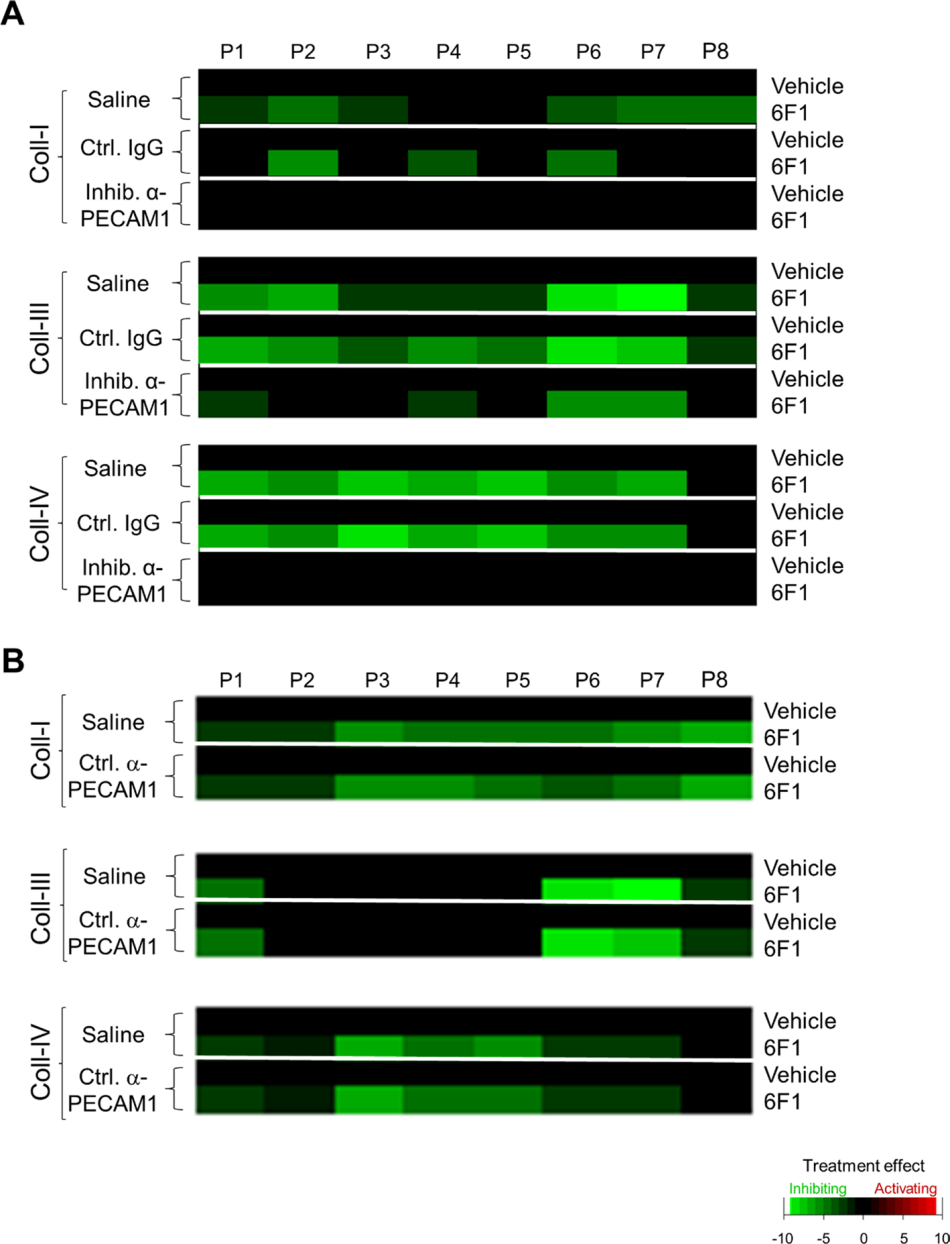
Selective effect of inhibitory anti-PECAM1 mAb in rescue of collagen-induced thrombus formation upon α2β1 inhibition. Blood samples were preincubated with 6F1 mAb (20 µg/mL) or vehicle, and then perfused during 3.5 min at 1000/s over microspots of collagen I, collagen III or collagen IV. Microspots were post-coated with saline, inhibitory anti-PECAM1 mAb (IgG1), mouse isotype control IgG1, or a control anti-PECAM mAb (clone MBC 78.2), as indicated. Multicolor microscopic images (EVOS-FL microscope, 60 × oil objective) were analyzed for eight parameters (Suppl. Table 1), which were univariate scaled. **A**, **B** Subtraction heatmaps for comparing per collagen type the effects of integrin α2β1 blockage (by 6F1 mAb) on parameters: P1, platelet deposition; P2, thrombus multilayer size; P3, thrombus morphological score; P4, thrombus multilayer score; P5, thrombus contraction score; P6, P-selectin expression; P7, integrin αIIbβ3 activation; P8, phosphatidylserine exposure. (A) Heatmapped effects of the 6F1 mAb on microspots of a collagen + inhibitory anti-PECAM1 mAb or the collagen + control IgG. **B** Heatmapped effects of 6F1 mAb on microspots of a collagen + control anti-PECAM1 mAb. Means ± SD (*n* = 3–7), tested for significance with one-way ANOVA. For color coding, a filter was applied of *p* < 0.05. Raw data are given in Suppl. Datafile 1

**Fig. 4:**
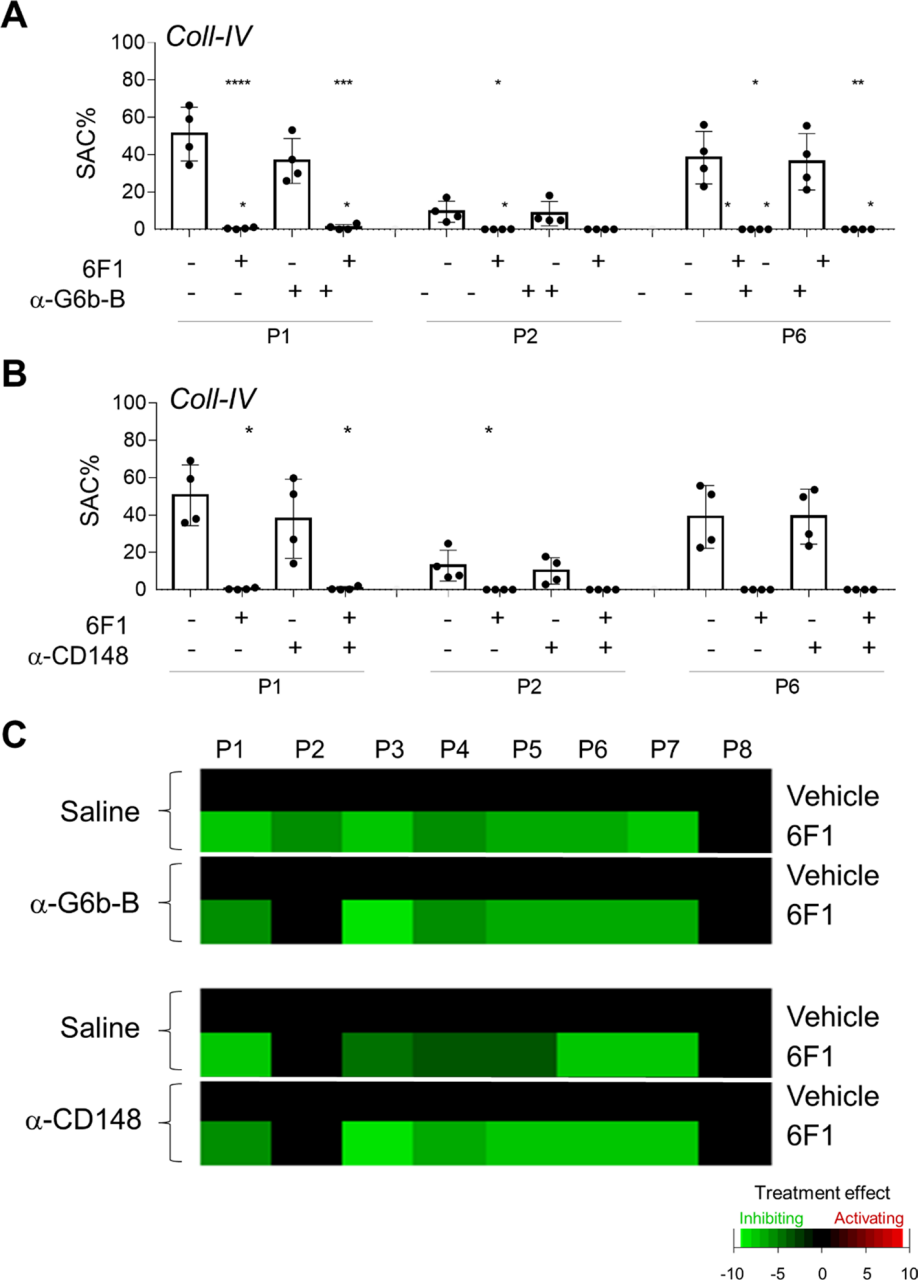
Inability of other antibodies against ITIM containing receptors to rescue collagen-induced thrombus formation upon α2β1 inhibition. Blood samples were preincubated with 6F1 mAb (20 µg/mL) or vehicle, and perfused during 3.5 min at 1000/s over microspots of collagen IV, as for Fig. 1. Microspots were post-coated with saline, anti-G6bB Ab (rabbit polyclonal) or anti-CD148 mAb (clone Ab1). Multicolor microscopic images, as for Fig. 1, were analyzed for eight parameters (Suppl. Table 1), which were univariate scaled. **A**, **B** Shown are quantified outcome data of collagen induced platelet deposition (parameter P1), thrombus multilayer size (parameter P2), and phosphatidylserine exposure (parameter P8) in the presence or absence of 6F1 mAb (in blood) and/or anti-G6bB Ab (**A**) or anti-CD148 mAb (**B**). In addition, subtraction heatmaps comparing the effects of integrin α2β1 blockage by 6F1 mAb on all 8 parameters (**C**). For color coding, a relevance filter was applied of *p* < 0.05. Means ± SD (*n* = 4), tested for significance with one-way ANOVA, *p < 0.05, **p < 0.005, ***p < 0.001 and ****p < 0.0001. Raw data are given in Suppl. Datafile 1

To confirm that the observed effects were confined to the inhibition of PECAM1, we repeated whole-blood flow experiments using collagen microspots that were co-coated with an isotype control antibody. From the collected images, again subtraction heatmaps were generated to compare the effects for 3 collagens and 8 parameters. First, the co-coating of collagens with mouse isotype control IgG1 did not alter the suppressive effect of α2β1 blockade for the majority of parameters (Fig. 3A). Second, collagen co-coating with another anti-PECAM1 mAb (clone MBC 78.2, mouse IgG), directed against the IgG domain 6 with activatory rather than inhibitory effect [34], similarly did not antagonize the effect of α2β1 blockage (Fig. 3B). Third, we investigated the co-coating with antibodies against the extracellular domain of other ITIM-linked receptors, namely G6b-B and CD148. This time, we focused on microspots of collagen IV, which gave the largest effects of α2β1 blockade. Neither a polyclonal anti-G6bB antibody (Fig. 4A) nor an anti-CD148 mAb (clone Ab1, inhibiting ITIM-mediated activity in bivalent form) [35] affected the parameters P1, P2 or P8 (Fig. 4B). Subtraction heatmaps confirmed the general lack of effect on all parameters P1-8 in the presence or absence of α2β1 (Fig. 4C).

As a final control, we checked if the inhibitory anti-PECAM1 mAb may have acted by platelet activation through the immunoglobulin FcγRIIA receptor [36]. Therefore, blood samples were pretreated with blocking anti-FcγRIIA mAb IV.3 at an effective concentration, and then flowed over collagen microspots with or without inhibitory anti-PECAM1 mAb. No significant effects of the IV.3 mAb were observed (Suppl. Fig. 2). We thus concluded that the rescuing effect of the inhibitory anti-PECAM1 mAb was independent of FcγRIIA-induced platelet activation.

In summary, these data indicated that the ability to recover the impaired platelet activation due to integrin α2β1 blockage was confined to the co-coating of collagens with inhibitory anti-PECAM1 mAb; and furthermore, that the recovery effects extended to α2β1 -dependent platelet adhesion to collagen I and IV. This suggested a mechanism of PECAM1- and ITIM-mediated suppression of collagen- and GPVI-induced thrombus formation, which becomes functional in the absence of α2β1-dependent platelet adhesion.

### Changes in collagen-induced thrombus formation in Noonan patients with* PTPN11* mutation

**Fig. 5:**
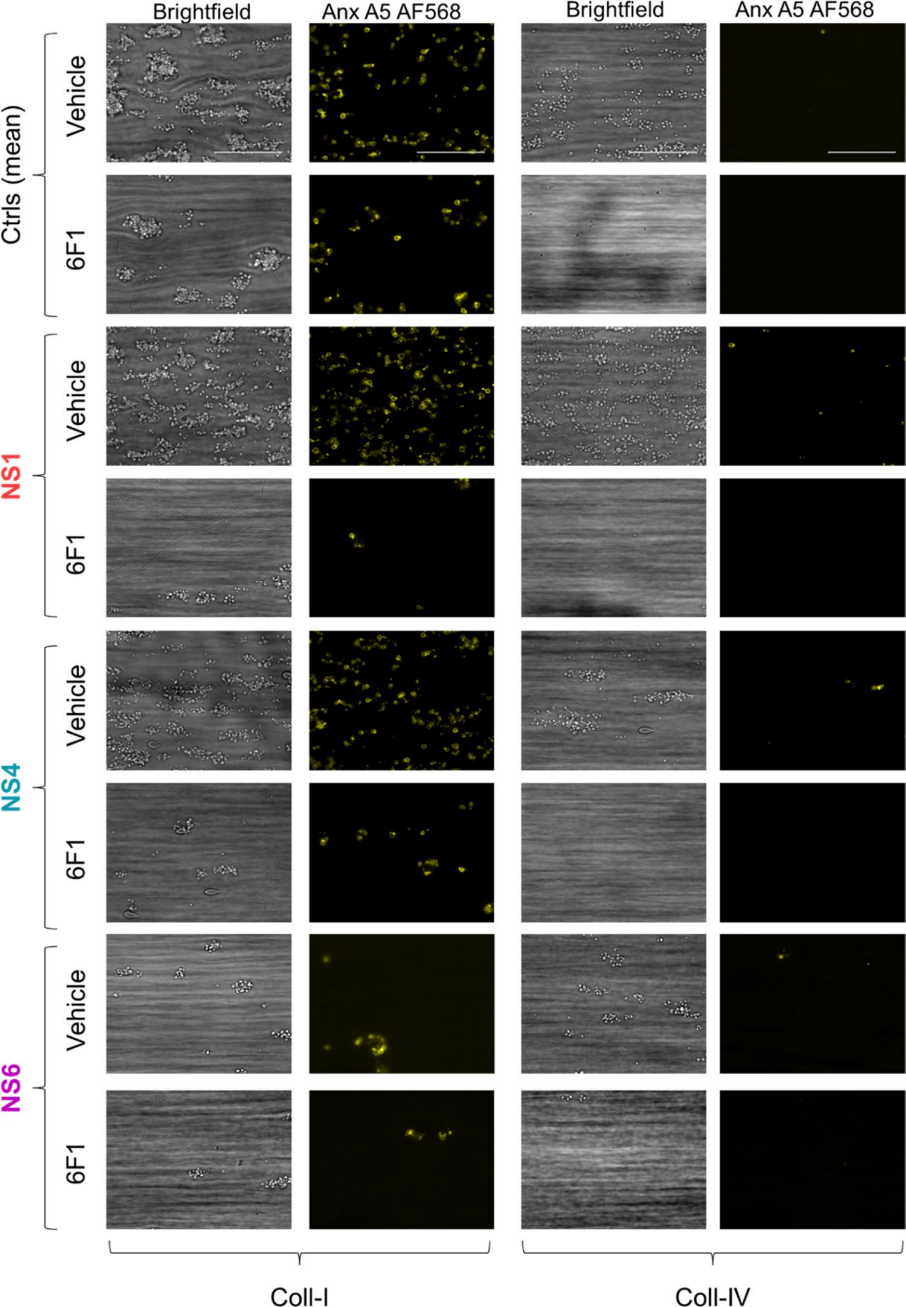
Alterations in collagen-induced thrombus formation for distinct Noonan syndrome patients. Whole blood from (day) control subjects (*n* = 13) and indicated patients (*n* = 6) was perfused at 1000 s^−1^ for 3.5 min over collagen surfaces (100 µg/mL), as described for Fig. 1. Collagen microspots were co-coated with saline or inhibitory anti-PECAM1 mAb (1 µg/mL). Blood samples contained vehicle solution or blocking α2β1 6F1 mAb (20 µg/mL). Shown are end stage brightfield (P1-5) and phosphatidylserine exposure (parameter P8) microscopic images obtained with blood from a representative day control subject and from patients NS1, NS4 and NS6 perfused over collagen-I or collagen-IV. As indicated, vehicle medium was present, or integrin α2β1 was blocked with 6F1 mAb. Scale bars, 50 µm

**Fig. 6:**
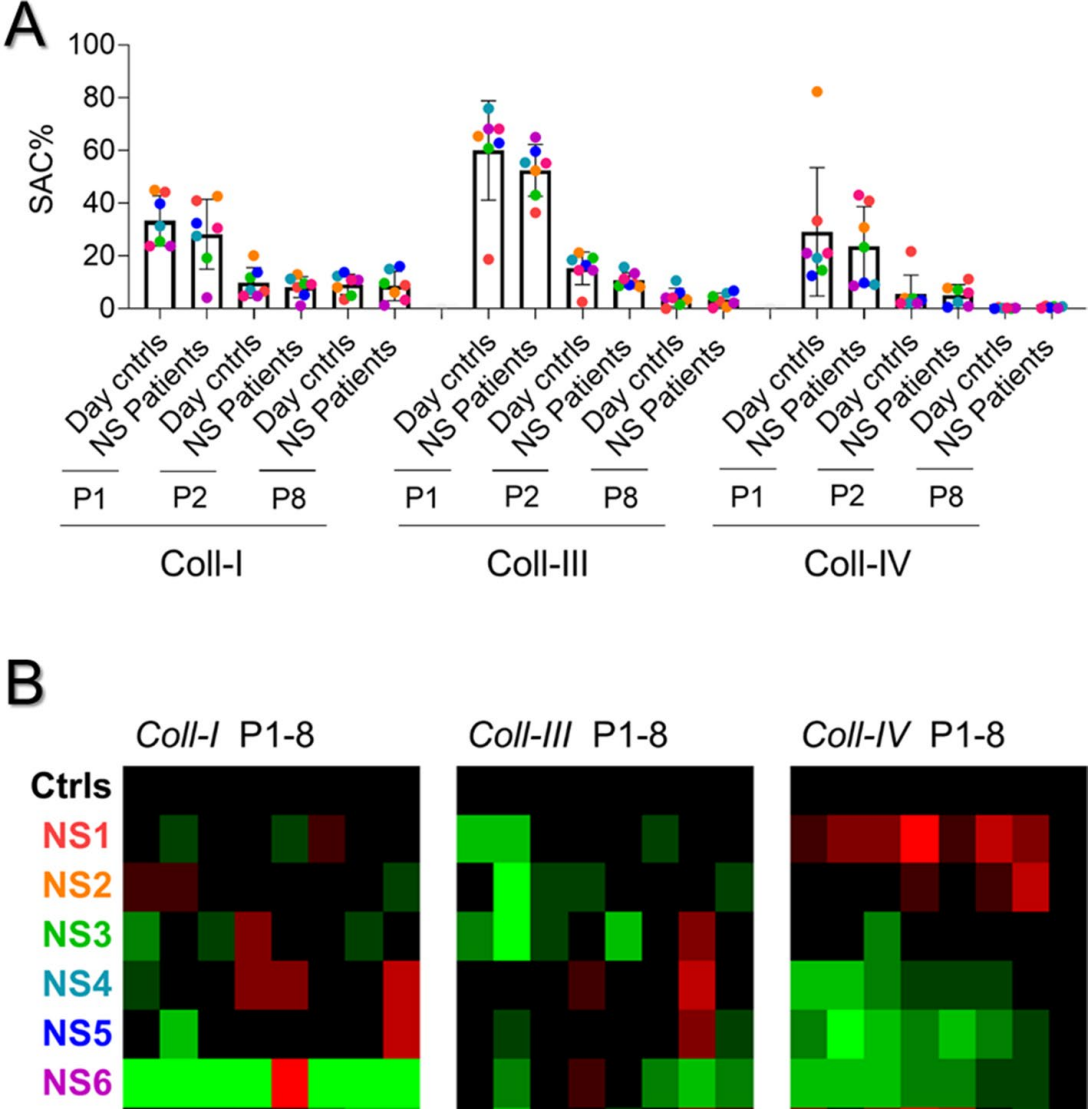
Alterations in collagen-induced thrombus formation with Noonan syndrome patients. Blood were analyzed from 13 control subjects, including 6 day controls, and 6 patients with Noonan syndrome (NS1-NS6), genotyped for a gain of function mutation in *PTPN11*. Blood samples were perfused over collagen I, collagen III or collagen IV, after which images were analyzed for 8 parameters (duplicate runs). **A** Quantification of platelet deposition (P1), thrombus multilayer size (P2), and phosphatidylserine exposure (P8) for individual day controls and patients per surface. **B** Heatmapped of scaled parameters per collagen type versus means of controls

In order to search for a role of PTPN11 signaling downstream of PECAM1, we collected blood samples from six Noonan syndrome patients (N1-6), all with a gain-of-function mutation in the *PTPN11* gene. The patients had normal platelet and red blood cell counts, while five of the patients had a (low) bleeding tendency (Table 1). After blood perfusion, we noticed heterogeneity of thrombus buildup between samples from individual patients, when compared to blood samples from day control subjects. Quantification of microscopic images indicated that the formation of platelet thrombi on collagen I and IV was relatively low for patients N4 and N6, but not for N1 (Fig. 5). After 0–10 scaling of all parameters and comparing the values per patient versus mean values ± SD of 13 control subjects, parameter sets of reduction were seen for patients N3-N6, which were strongest for patients N3 to N6 on collagen IV, and for N6 extended to collagen I (Fig. 6A, B).

**Table 1:** Overview of healthy subject and genotyped patients hematological parameters

Subject	Age (years)	Red blood cells (10^12^/L)	Platelets (× 10^9^/L)	Bleeding score	Genetics *PTPN11*
Day controls (6)*	> 22	4.15 ± 0.33	272 ± 79	n.d.	n.d
NS1	38	3.70	245	13	c.188A > G
NS2	13	4.78	212	1	c.1510A > G
NS3	12	4.79	148	1	c.205G > C
NS4	13	4.98	266	0	c.1510A > G
NS5	13	5.03	206	2	c.922A > G
NS6	5	4.93	254	0	c.1507G > A

*NS*, confirmed Noonan syndrome with (likely) pathogenic variants of *PTPN11*

*Mean ± SD

**Fig. 7:**
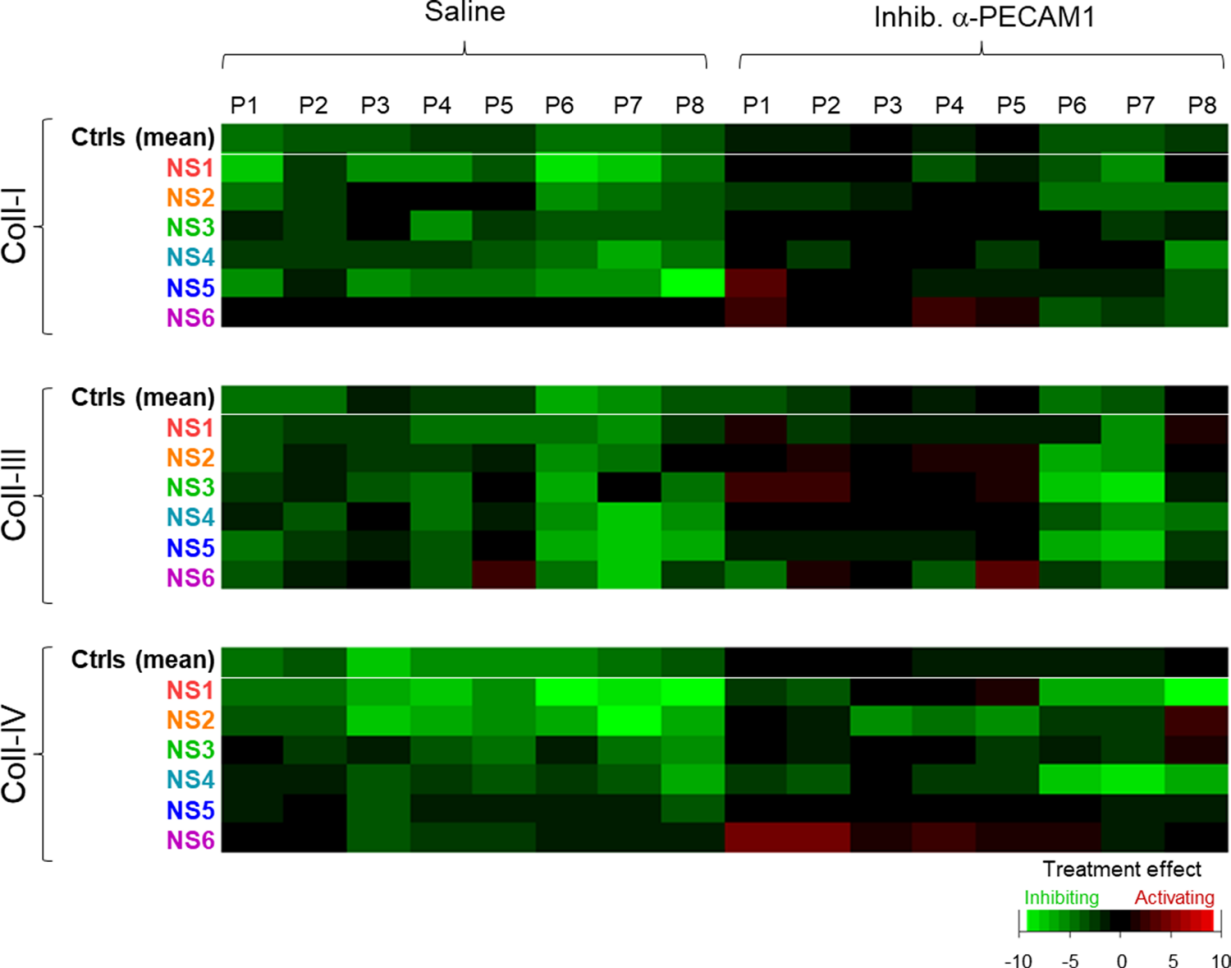
Increased responsiveness to α2β1 blockage with Noonan syndrome patients Parameters of thrombus formation for 13 controls subjects and 6 patients were recorded as for Fig. 6. Collagen microspots contained vehicle or inhibitory anti-PECAM1 mAb; and blood flow was in the presence or absence of 6F1 mAb. Shown is a subtraction heatmap of 6F1 mAb effects (scaled parameters P1-8) per patient for collagen I, collagen III and collagen IV containing or not inhibitory anti-PECAM1 mAb. Means ± SD (*n* = 13 controls). For raw data, see Suppl. Datafile 1

To determine the sensitivity of the patient platelets for α2β1 blockage, additional flow runs were performed in the presence of 6F1 mAb, with collagen spots downstream co-coated with the inhibitory anti-PECAM1 mAb. A subtraction heatmap again was made to assess the 6F1 mAb effects in the presence or absence of anti-PECAM1 mAb per patient, such in comparison to averages in the control cohort. This analysis showed for all three collagen surfaces a large reducing effect of α2β1 inhibition for the patient blood samples, which was strongest for patients N1-N2 with a relatively high thrombus formation (Fig. 7, left part). Markedly, for 5 of the 6 patients (collagens I and IV), the reducing effect of α2β1 inhibition was reverted upon blockage of PECAM1, thus resulting in a normalization of the thrombus-forming process (Fig. 7, right part). Together, this suggested that the gain-of-function of PTPN11 in the majority of patients led to a lower collagen-induced platelet activation, which still relied on platelet adhesion via α2β1, but became normalized by PECAM1 inhibition upon absence of α2β1.

## Discussion

In the present paper, we assessed to which extent the ITIM-linked receptor PECAM1 and its connected tyrosine phosphatase are able to suppress collagen-induced thrombus formation mediated by the two collagen receptors, GPVI and integrin α2β1. To investigate this, we perfused whole blood over collagen types I, III and IV, with a high or low GPVI-activating potential, respectively [31]. The results pointed to a thrombus-suppressing effect of PECAM1 that was independent of the collagen type and most prominent under conditions of α2β1 blockage. This thrombus-modulating effect extended to Noonan patients with a gain-of-function mutation in the ITIM-linked tyrosine phosphatase PTPN1, several of whom also showed clear impairments without α2β1 blockage.

For over two decennia, it is known that the PECAM1 receptors on human or mouse platelets can restrict and modulate platelet responses evoked by collagens [37, 38]. One report has also shown that PECAM1 acts as a negative modulator of laminin-induced platelet activation [39]. Furthermore, there is evidence that PECAM1 activity negatively regulates platelet responses to agonists of G protein-coupled receptors [12, 16]. Other reports using knockout mice pointed to a weak role of PECAM1 in terms of GPVI-induced platelet activation [40], but a larger role in megakaryocyte development [41]. In our experiments, we found that a negative regulatory contribution of PECAM1 to collagen-dependent thrombus formation became prominent upon blockage of integrin α2β1, and was surprisingly independent of the type of collagens, *i.e.* with high or low GPVI-induced platelet responses. As selective tool in the studies, we used the inhibitory anti-PECAM mAb WM59. This antibody recognizes the first or second Ig domain of PECAM1, and thereby prevents its homophilic ligation and consequently the signaling via PECAM1 [16]. By contrast, an antibody against the sixth Ig domain of PECAM1, which increases rather than inhibits PECAM1 activity [15, 42], did not affect thrombus parameters. Jointly, our results point to an overall weak role of PECAM1 in collagen-induced platelet activation, which however becomes most prominent under conditions of low or blocked integrin involvement. In other words, the data indicate that the suppression of GPVI-dependent thrombus formation by either PECAM1 or a gain-of-function of PTPN11 is overruled by α2β1 engagement.

Estimates for human platelets indicate that PECAM1 on average is expressed at similar levels (9,435 copies/platelet) as GPVI (9577 copies), integrin α2β1 (4588 copies) and PTPN11 (3666 copies) [20]. Common understanding is that PECAM1 as a receptor uses another PECAM1 molecule as ligand, although also other ligands are described, such as integrin αvβ3 and the immune cell receptor CD38 [43, 44]. The here identified role of PECAM1 on platelet activation under flow as a function of α2β1-dependent platelet adhesion likely is most relevant in situations of low α2β1 expression or function. Regarding this platelet integrin, known are three single nucleotide variants of the *ITGA2* gene, encoding for the α2 chain (807C/T, 1648A/G and 2531C/T) which together explain the up to tenfold inter-individual variation in platelet α2β1 expression[45–47]*.* Association studies link these variants to bleeding [48] or thrombosis risks [47, 49], with variable evidence for alteration in classical, stirred collagen-induced platelet aggregation [49, 50]. In addition, the 219A/G variant of *GP6*, explaining 20% of the platelet GPVI level, co-determines the extent of collagen-dependent thrombus formation [51], and links to arterial and venous thrombosi*s* [52]. A functionally relevant variant of PECAM1 (rs1867624) has also been reported [53], but the link to platelet function is unknown.

In the past decades, few unrelated patients with defective (acquired) integrin α2β1 expression, mild thrombocytopenia and a mild bleeding tendency have been described [54–56]. This phenotype is commonly defined as bleeding disorder platelet-type 9 (BDPLT9, OMIM: 614,200). At that time, it was postulated that in some of the patients other platelet receptors may have functionally masked the integrin deficiency [56]. In our study, we did not measure the levels of collagen receptors or PECAM1 on platelets from healthy blood donors or Noonan patients, but it can be envisioned that a PECAM1-dependent restraint of thrombus formation is physiologically most relevant for particular (genetic) combinations, e.g. high GPVI, high PECAM1/PTPTN11 and low α2β1. To establish such links of multi-receptor genetic variance with platelet function is will require further studies.

To investigate the likely tyrosine phosphatase involved, we used blood from patients with Noonan syndrome and an established gain of function mutation in the *PTPN11* gene, which encodes for the corresponding protein tyrosine phosphatase non-receptor (also known as SHP2). Noonan patients with such mutations present with a variable bleeding diathesis [25], which associated with a partial impairment in collagen-dependent thrombus formation [24]. Importantly, using blood from four out of six patients, we also observed reductions in thrombus parameters, which partly extended to all collagens. Interestingly, for 5 of the 6 patients, in the presence of α2β1 blockade, we noticed a normalization of the thrombus formation by PECAM1 inhibition, in comparison to control subjects. This suggested that a gain of function of PTPN11—via altered signaling or protein expression mechanisms in the patient platelets—negatively modulates the thrombus formation on collagens, particularly under conditions where α2β1 is inactive.

Our data extend the evidence from other papers, that under flow conditions, synergy between GPVI and α2β1 determines the extent of platelet activation and aggregation [6, 8, 9, 30]. We suppose that the phosphatase-linked PECAM1 is a co-regulatory element enforcing GPVI activity on the level of signal transduction under conditions, where platelet-collagen interaction via the integrin is weakened on the receptor occupancy level. Indirect evidence for such a mechanism comes from studies that PECAM1 suppresses platelet activation on laminin, e.g. a low integrin-dependent adhesive protein [39]. This leads to the concept that in flow-dependent platelet activation PECAM1 has an intrinsic ITIM-dependent tyrosine phosphatase activity (involving PTPN11), which becomes functional when the α2β1-dependent stabilization of platelets at GPVI epitopes on collagens is diminished. Pathophysiologically relevant conditions will be situations of low integrin–collagen interaction (including low integrin expression), altered PTPN11 activity such as in Noonan syndrome patients, and partial interference in platelet GPVI activity.

Taken together, our results indicate that the PECAM1 and PTPN11 restraining mechanisms on collagen-induced thrombus formation are partly dependent of the extent of GPVI activation, and also on other factors such as the engagement of α2β1. Accordingly, the present work underlines the relevance for overlapping functions of the platelet collagen receptors and the potential of inhibitory signaling pathways to rescue defective phenotypes. Collectively, these results indicate that restraining mechanisms by PECAM1 and PTPN11 on thrombus formation are most operative: at low GPVI-activating collagens and in the absence of α2β1 engagement.

### Supplementary Information

Below is the link to the electronic supplementary material.

Supplementary file1 (DOCX 1165 KB)

Supplementary file2 (XLSX 215 KB)

## Data Availability

The data supporting the results of this article are provided in the on-line Supplementary Datafile 1.
