# Supplementary material for: Restraining of glycoprotein VI- and integrin α2β1-dependent thrombus formation by platelet PECAM1
Source: Cell Mol Life Sci. 2024 Jan 18;81(1):44. doi: 10.1007/s00018-023-05058-2 (PMC10796532; doi:10.1007/s00018-023-05058-2)
Supplement: Supplementary file 1 — Supplementary file1 (DOCX 1165 KB) [file 18_2023_5058_MOESM1_ESM.docx]

**Supplementary Material**

**Restraining of glycoprotein VI- and integrin α2β1-dependent thrombus formation by platelet PECAM1**

Natalie J. Jooss,^1,2^ Marije G. Diender^3^ Delia I. Fernández,^1,4^ Jingnan Huang,^1,4^ Floor C. J. Heubel-Moenen,^5^ Arian van der Veer,^3,6^ Marijke J.E. Kuijpers,^1^ Natalie S. Poulter,^2,7^ Yvonne M.C. Henskens,^8^* Maroeska te Loo^3^*, Johan W.M. Heemskerk^1,9^*

^*authors contributed equally^

**Supplementary Table**

**Supplementary Table 1. Annotations of microspots and parameters of thrombus formation.** Indicated per microspot are contributions of receptors to whole blood thrombus formation, i.e. GPIb-V-IX, GPVI and integrin α2β1 (o, +, ++, ++), adapted from Refs. ^1,2^ Furthermore, listing of image analysis parameters (P1-8) from brightfield and fluorescence microscopy, and factor used for univariate scaled heatmaps. N.d., not determined.

**Microspot GPIb-V-IX GPVI α2β1**

M1 collagen I (± Ab post-coating)*) + ++ +

M2 collagen III (± Ab post-coating)F*) ++ + +

M3 collagen IV (± Ab post-coating)*) + + +

**Parameter Range Scaled**

*Platelet adhesion (brightfield)*

P1 platelet deposition (% SAC) 0 - 66 0 - 10

*Thrombus phenotype (brightfield)*

P2 thrombus multilayer size (% SAC) 0 - 19 0 - 10

P3 thrombus morphological score 0 - 4 0 - 10

P4 thrombus multilayer score 0 - 3 0 - 10

P5 thrombus contraction score 0 - 3 0 - 10

*Platelet activation (fluorescence)*

P6 P-selectin expression (AF647 αCD62P mAb, % SAC) 0 - 56 0 - 10

P7 integrin αIIbβ3 activation (FITC α-fibrinogen Ab, % SAC) 0 - 24 0 - 10

P8 PS exposure (AF568-annexin A5, % SAC) 0 - 10 0 - 10

References:

**1.** Jooss NJ, De Simone I, Provenzale I, et al. Role of platelet glycoprotein VI and tyrosine kinase Syk in thrombus gormation on collagen-like surfaces. *Int J Mol Sci.* 2019;20:2788.

**2.** van Zanten GH, de Graaf S, Slootweg PJ, et al. Increased platelet deposition on atherosclerotic coronary arteries. *J Clin Invest.* 1994;93:615-632.

**Supplementary Figures**

**
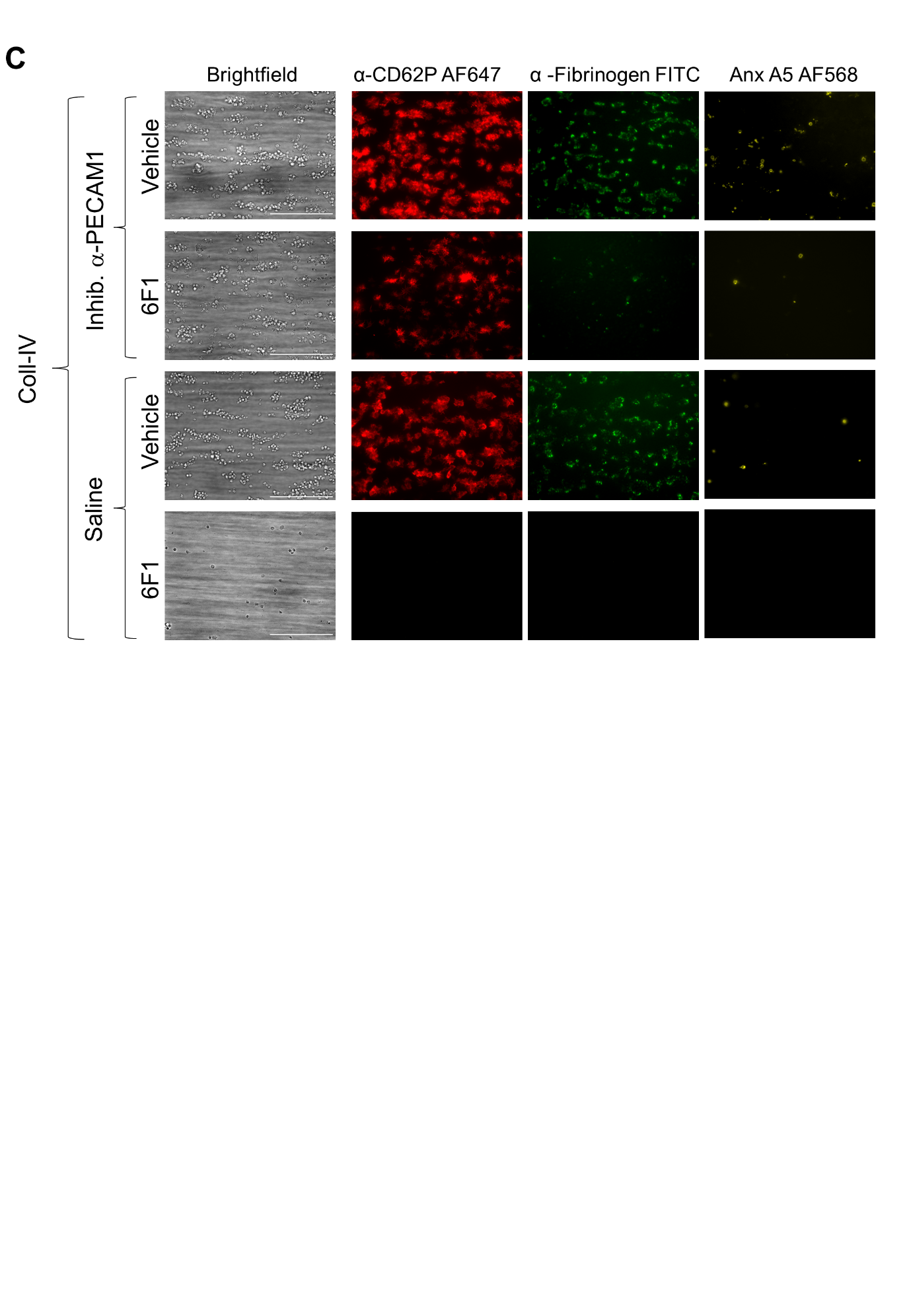
**

**Suppl. Figure 1. Enhanced collagen induced thrombus formation by PECAM1 blockage in the absence of integrin α2β1: representative microscopic images.** Blood samples were preincubated with anti-α2β1 6F1 mAb (20 µg/mL) or vehicle medium, and then perfused during 3.5 minutes at 1000 s^-1^ over microspots of collagen-IV, post-coated with saline or inhibitory anti-PECAM1 mAb (WM59). See also Figure 1. Shown are representative images for per collagen surface with or without inhibitory anti-PECAM1 mAb (n=6-7). Scale bars, 50 µm.


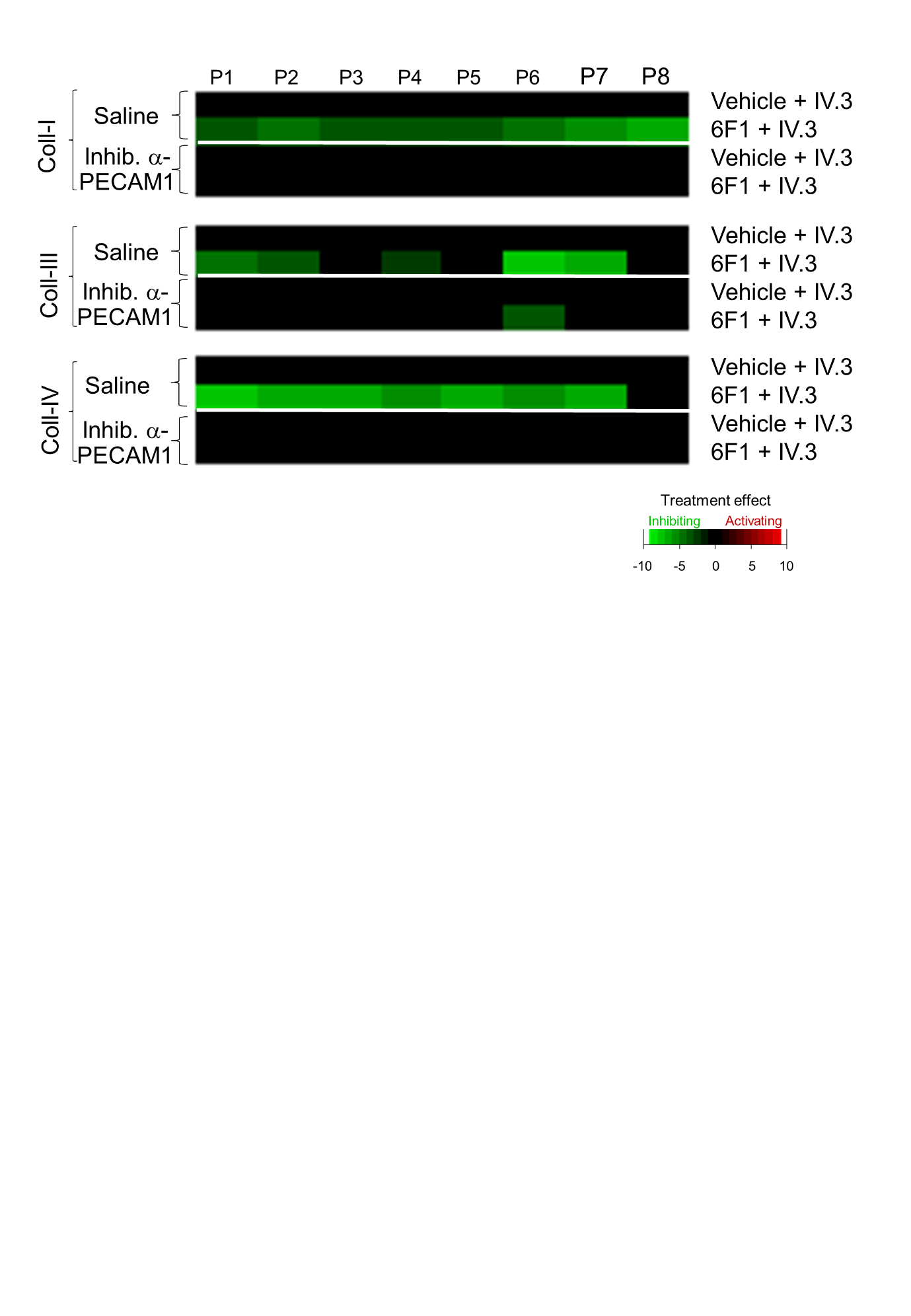


**Suppl. Figure 2. Absence of FcγRIIA role in anti-PECAM1 antibody effect on thrombus formation.** Blood samples were preincubated with inhibitory anti-FcγRIIA mAb (20 µg/mL) with or without anti-α2β1 6F1 mAb (20 µg/mL) and vehicle medium, and then perfused during 3.5 minutes at 1000 s^-1^ over microspots of collagen-I, collagen-III or collagen-IV, as for Figure 1. Microspots were post-coated with saline or inhibitory anti-PECAM1 mAb (WM59), as indicated. Multicolor microscopic images were analyzed for eight parameters, which were univariately scaled. Shown is a subtraction heatmap, comparing per collagen type, the effects of combined integrin α2β1 (6F1 mAb) and FcγRIIA blockage (by IV.3 mAb) for parameters P1-8. Means ± SD (n=3), tested for significance with one-way ANOVA. For color coding, a filter was applied of *p*<0.05. Raw data for P1-8 are given in the Supplemental Datafile.
